# Marimo machines: oscillators, biosensors and actuators

**DOI:** 10.1186/s13036-019-0200-5

**Published:** 2019-09-03

**Authors:** Neil Phillips, Thomas C. Draper, Richard Mayne, Andrew Adamatzky

**Affiliations:** 1 Unconventional Computing Laboratory, University of the West of England, Coldharbour Lane, Bristol, BS16 1QY UK; 2 Department of Applied Sciences, University of the West of England, Bristol, BS16 1QY UK

**Keywords:** Marimo, Processor, Logic gate, Oscillator, Biosensor, Bioactuator, Biomotor, Algae, Unconventional Computing, Biomimicry, TRIZ

## Abstract

**Background:**

The green algae balls (*Aegagropila linnaei*), known as Marimo, are large spherical colonies of live photosynthetic filaments, formed by rolling water currents in freshwater lakes. Photosynthesis therein produces gas bubbles that can attach to the Marimo, consequently changing its buoyancy. This property allows them to float in the presence of light and sink in its absence.

**Results:**

We demonstrate that this ability can be harnessed to make actuators, biosensors and bioprocessors (oscillator, logic gates). Factors affecting Marimo movement have been studied to enable the design, construction and testing of working prototypes.

**Conclusions:**

A novel actuator design is reported, incorporating an enhanced bubble retention system and the design and optimisation of a bio-oscillator is demonstrated. A range of logic gates (or, and, nor, nand, xor) implementable with Marimo have been proposed.

**Electronic supplementary material:**

The online version of this article (10.1186/s13036-019-0200-5) contains supplementary material, which is available to authorized users.

## Background

Increasingly scientists and engineers are realising that nature, over many millions of years of evolution, has by necessity developed energy efficient systems. Combining some of nature’s biochemical processes with human engineering (biomimetic) has enabled novel devices to be prototyped [1].

The variability in living systems, bred from evolutionary pressures, can take the form of movement with minimal energy consumption. In plants, responses that occur in response to a directional stimulus are called tropisms [2]. One of the most commonly observed tropic responses in plants is phototropism, in which plant stems grow towards light. Heliotropism is the basis for rapid and reversible movement in plants, which allows them to track the sun. Plants can also exhibit non-directional responses to stimuli (nastic movements), for example temperature and humidity. The rate or frequency of these responses increases as intensity of the stimulus increases [3].

Nature has adapted to harness solar energy via photosynthesis, which provides the basic energy source for nearly all organisms. The maximum photosynthetic efficiency of algae is an active topic of debate [4]. The theoretical potential (approximately 36% [5]) cannot be achieved in practice due to limitations of the relevant bio-chemical processes. At low light intensity algae can convert roughly 6% of Photosynthetically Available Radiation (PAR) into biomass in a best case scenario [6]. This conversion rate drops in full sunlight to avoid damaging the organism [7].

**Fig. 1:**
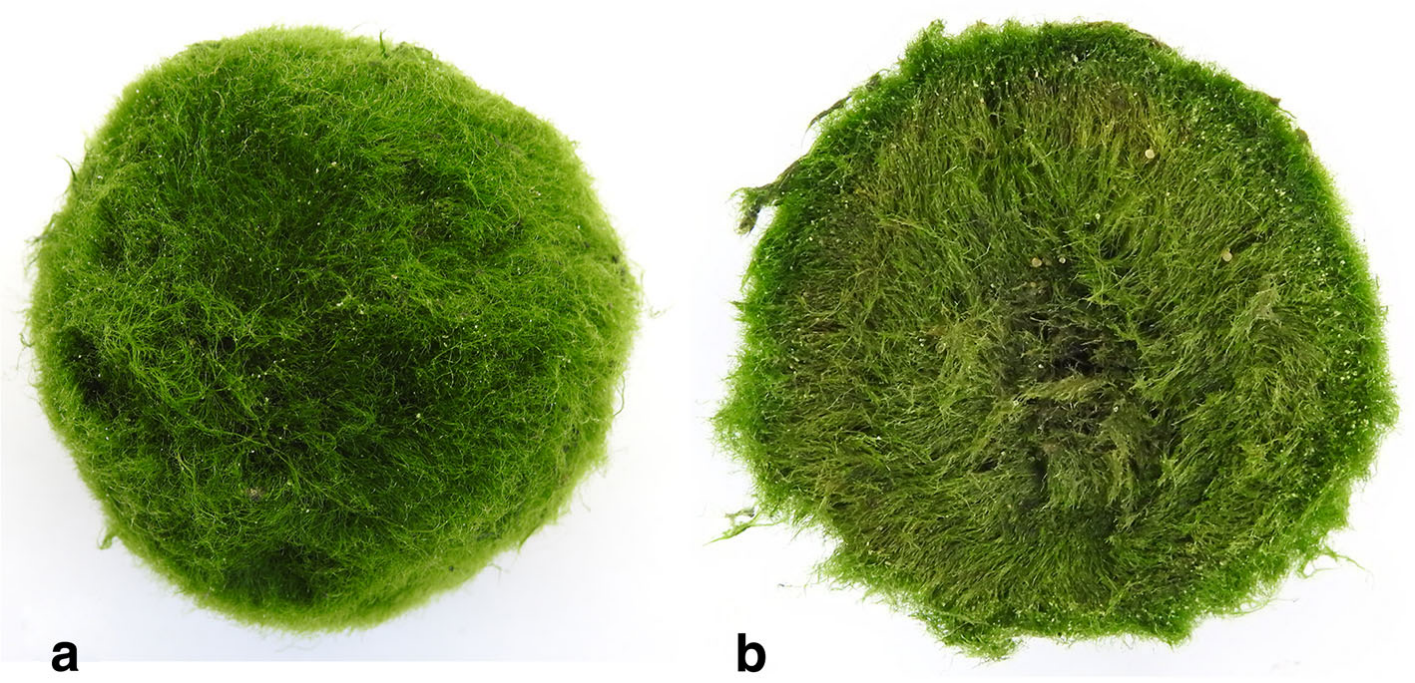
Photographs of an (**a**) intact and (**b**) cross-sectioned Marimo. Small grains of sand are visible in both images. The diameter of the Marimo is 62mm

This investigation focuses on harnessing adaptive responses of a live heliotropic, photosynthetic organism for use in hybrid bio-artificial systems. *Aegagropila linnaei* balls are large (exceeding tens of centimetres in diameter in some cases) spherical objects [8, 9] formed by the natural rolling and self-adhesion of filamentous alga over many years in turbulent freshwater lake currents [10, 11]. *A. linnaei* are known more commonly (and hereafter in this paper) by the Japanese monicker “Marimo”, from the ubiquity of the alga balls arising from Lake Akan, Hokkaidō, Japan [12, 13]. Photographs of both an intact Marimo and the cross-section of a Marimo can be seen in Fig. 1a and Fig. 1b, respectively. In the cross-sectional photograph, it can be seen that the filamentous nature of the Marimo is continuous throughout. Additionally the outer edge is a darker green than the core, which is believed to be due to the photosynthetic pigment concentrating in the regions that receives the most illumination, in agreement with previously published works [10].

After considering a range of algae structures it was concluded that Marimo was particularly promising for utilisation in functional bio-artificial devices. Marimo can grow in three forms: (1) epilithic, usually found on the shaded side of rocks; (2) free-floating filaments, that can form a carpet on the surface of the water; and (3) densely packed algal filaments, that radiate from the centre forming spherical shape [14]. For our purposes, the latter has the advantages of being self-contained, mobile, and able to photosynthesise using light from any direction [15]. Furthermore, Marimo appear to have an extraordinarily long lifespan, with literature citing that natural balls are formed over ‘many years’ [10] and commercial suppliers advertising prised ornamental specimens over 10 cm in diameter, which are reportedly grown over a period of 15 or more years. This suggests a long lifespan of any proposed bio-artificial constructs.

Other researchers [16] have studied the natural characteristics of Marimo; in particular, its ability to rise and sink in water, which was found to result from generation of oxygen via photosynthesis. Bubbles are formed on the surface of, and at shallow depths within, the Marimo when they are provided with illumination: it is assumed that the filamentous nature of the alga both provides numerous nucleation sites and creates a mesh through which it is difficult for the oxygen bubbles to dissipate. The observed phenomenon of a Marimo ball rising when provided with a means to initiate photosynthesis suggests that the oxygen generation, and retention as bubbles adherent to and within the moss balls, may exceed the rate at which oxygen is lost through dissipation or percolation through its filamentous structure.

Several research groups have reported on bioenergy, usually through converting biomass into electricity or secondary products [6]. Other groups have reported biomimetic microsystems with buoyancy control using features such as: Pt:Ag microbeads decomposing H_2_O_2_ [17], clay-coated catalase-containing microcapsules which decompose H_2_O_2_ [18], or metal-organic frameworks containing catalase for the decomposition of H_2_O_2_ [19]. However, using Marimo to *directly* power processors, bio-sensors and actuators through exploitation of its photosynthetic ability has yet to be explored. The research reported here represents a step towards the long term goal of autonomous, light powered, biological systems which can operate under real world conditions. To expand on the benefits of using biological components for engineering and computing applications, many characteristics of biological systems can be considered as desirable if exploited for a useful task, such as self-growth, low energy consumption, carbon capture (in photosynthetic organisms), organisation and variation. This ethos is predicated on minimising the use of conventional electronics, as bio-artificial hybrid devices necessarily exhibit the drawbacks of both types of material. Therefore, biological devices are not considered as direct replacements for their artificial counterparts (e.g. as ‘biological time’ is slower than electrical communications, biological solutions are typically not suited to time-critical applications), but as complementary systems.

Rather than using the biomass formed from the photosynthesis, we took the unconventional approach of utilising the gas generated during the photosynthetic process instead. More specifically, the low density of the gas (0.001g cm ^−3^) compared to water (1.0g cm ^−3^) means the gas rises in the form of bubbles to minimise overall Potential Energy (PE). The movement of the bubbles towards the surface of the water can be harnessed to enable a variety of systems.

We demonstrate that a variety of actuating and, potentially, computing devices can be implemented by using Marimo with a controlled patterns of illumination. We propose experimental designs of Marimo motor and oscillator and speculate about potential implementation of Marimo logic gates.

## Methods and materials

### Marimo culture

Artificially-rolled Marimo were sourced from Amazon UK and were kept in large, lidded aquariums in tap water containing 0.1mL L ^−1^ of commercially-available fertiliser containing a source of phosphates and nitrates (TNC Complete, The Nutrient Company). When not in use in experiments, the organisms were exposed to a day/night cycle but were kept out of direct sunlight. Aquarium water and fertiliser was refreshed every 3 weeks. All Marimo culture and consequent experimentation was conducted at room temperature (18 ^∘^C to 22 ^∘^C). For a detailed measurement of illumination level, a Photosynthetically Active Radiation (PAR) sensor was used (see Additional file 1 for bubble, light and calibration data).

### Bubble retention

Cohorts of Marimo (60 mm diameter) were monitored under the same conditions (light and nutrients). All were observed to produce bubbles, but only some gained sufficient buoyancy to raise off the floor of the aquarium and float. The proportion of bubbles which stay attached to algal filaments is noted to affect overall buoyancy. In other words, bubble generation *and* retention is required to achieve flotation. The distribution of bubbles on the surface (of undisturbed) water above Marimo was monitored. The density of bubbles towards the periphery doesn’t increase in proportion to Marimo surface area which suggests it is easier for the bubbles to escape from the algal filaments when their lift (due to buoyancy) is upwardly aligned with the filaments.

### Buoyancy increase from bubbles

The positive buoyancy provided by bubbles was measured by carefully adding weights to floating Marimo until they sunk. To minimise unwanted rotation and associated loss of bubbles the weights were formed from rings of copper wire with a diameter smaller than the diameter of the Marimo. The typical lift provided by bubbles on Marimo of approximately 60 mm diameter is 1.5 g. The surface area of a 60 mm diameter Marimo is 110 cm^2^. However, only part of the bottom hemisphere usually holds larger bubbles, so the effective lift area is 50 cm^2^. Therefore, usable lift is approximately 0.03g cm ^−2^. To investigate the influence of illumination on bubble generation Marimo were located under (water filled) glass funnels with attached syringes, such that any released bubbles would rise inside the funnel and be captured. The change in volume of air inside the syringes (50 mL) was recorded while maintaining the same water level inside the funnel. One beaker was intentionally left empty and acted as ‘control’ (no gas was collected). Five funnel rigs were left running (simultaneously) for two weeks. To ascertain the influence of the concentration of dissolved carbon dioxide on photosynthesis, a Marimo was continuously illuminated (Martin Rush Light Source, see Additional file 1) for a prolonged period (10 days) in still water until bubble formation (photosynthesis) was observed to stop. Once ceased, frozen carbon dioxide (dry ice, 8 g) was added to elevate the concentration of carbon dioxide in the water.

### Marimo motor

**Fig. 2:**
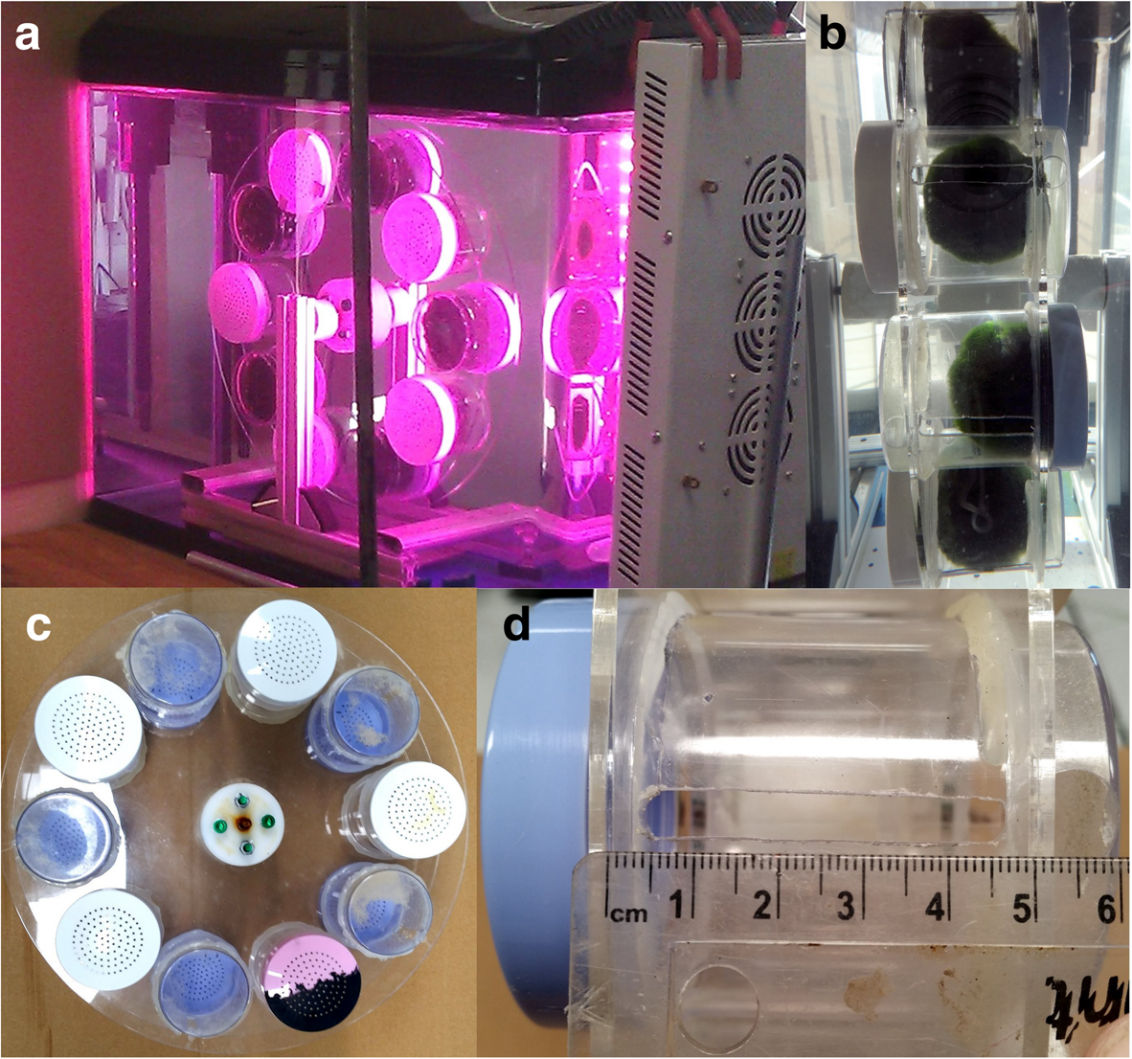
Marimo motor. **a** Motor in aquarium and LED lamp. **b** Side view of Marimo in plastic pots. **c** Plan view of rotor assembly. **d** Bubble escape slot. A video of the Marimo motor rotating can be seen in the Additional file 3

Ten Marimo (approximately 60 mm diameter) were located inside transparent chambers (70 mm diameter, 85 mm length, with screw cap lids) equally spaced around the periphery of two transparent, acrylic discs (340 mm diameter), see Fig. 2c. The discs were mounted on bespoke ‘friction-free’ magnetic bearings (see Additional file 1). The rotor assembly was dynamically balanced (see Additional file 1) to within 50 g mm by adding correcting weight to both discs as required. Alternatively, an automatic balancing set-up could be exploited [20]. The performance of the motor was separately investigated with sunlight and illumination solely from an LED growth light (120 W input) on the side of the aquarium, see Fig. 2a. The aquarium water re-circulation pump was left switched off to prevent recirculating water affecting measurements. A camera (Nikon P900) was programmed to take a photo every four minutes, in order to determine the speed and direction of rotation. Testing revealed the location and size of the bubble release slots has a significant impact on speed and smoothness of rotation. Slots 6mm wide and 40mm in length were used, through trial and error, see Fig. 2b and Fig. 2d.

### Marimo oscillator

**Fig. 3:**
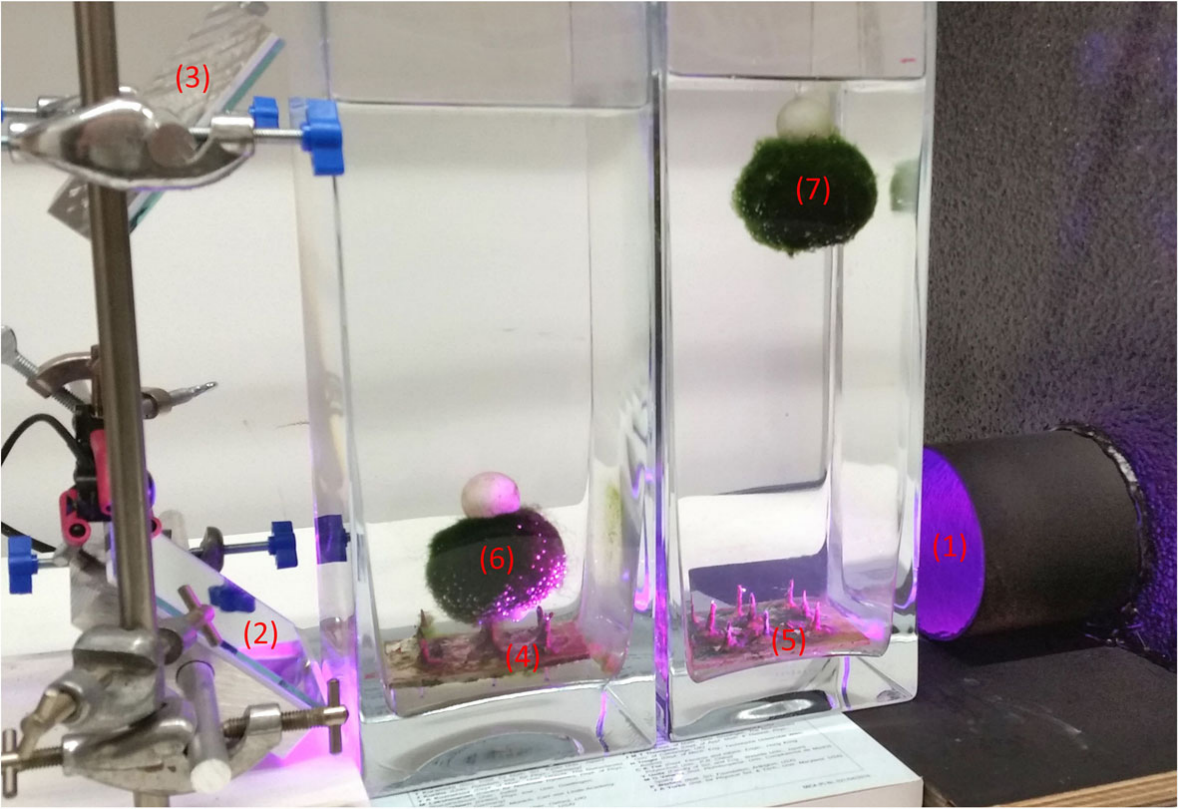
Experimental rig of Marimo oscillator. (1) parallel light beam via matt black tube from LED lamp, (2, 3) mirrors, (4, 5) spikes and (6, 7) Marimo with floats and weights

Two Marimo (approximately 60 mm diameter) were place in transparent columns of water (110 ×110×300mm, see Fig. 3), free to move up and down when illuminated. The ‘right hand’ Marimo (nearest the lamp) blocks light reaching the ‘left hand’ Marimo. The illumination (from Martin Rush LED lamp, set to 18% red, 18% blue and 0% green, see Additional file 1) was made more parallel by passing through tube (80 mm diameter and 200 mm length) with matt black surface. After a period of illumination the ‘right hand’ Marimo floats and light illuminates the ‘left hand’ Marimo, which then floats. The light beam is then reflected by a pair of mirrors onto the floating ‘left hand’ Marimo. The ‘right hand’ Marimo then sinks, blocking the light to the ‘left hand’ Marimo. This then causes the ‘left hand’ Marimo to sink. This oscillating cycle repeats continuously. In order to restrict Marimo movement to the vertical axis, spikes were added to the bottom of the water columns. The acrylic bases of the spikes were coated in silicon to reduce nucleation sites for bubble formation. The centre of mass of the Marimo was lowered as discussed in the “Results” section. Additionally, the level of the water in the columns was set such that, whilst floating, illumination to bottom half of the Marimo was most prominent. The experimental set up and diagram of the oscillations can be seen in Fig. 3. A bespoke parallel (4 ^∘^) light beam (660 nm) was developed to enhance performance (see Additional file 1).

## Results

### Buoyancy & movement enhancement

It was observed the typical rate of bubble generation (for approximately 60 mm diameter Marimo) in moderate sun-light is 1 mL d ^−1^ to 3 mL d ^−1^. The rate can increase to 6 mL d ^−1^ in strong sunlight. After a prolonged period of illumination, the bubble production of the Marimo was observed to decrease. After addition of dry ice, no additional generation of bubble formation was observed, suggesting that the carbon dioxide concentration is not responsible for the decrease in photosynthesis.

**Fig. 4:**
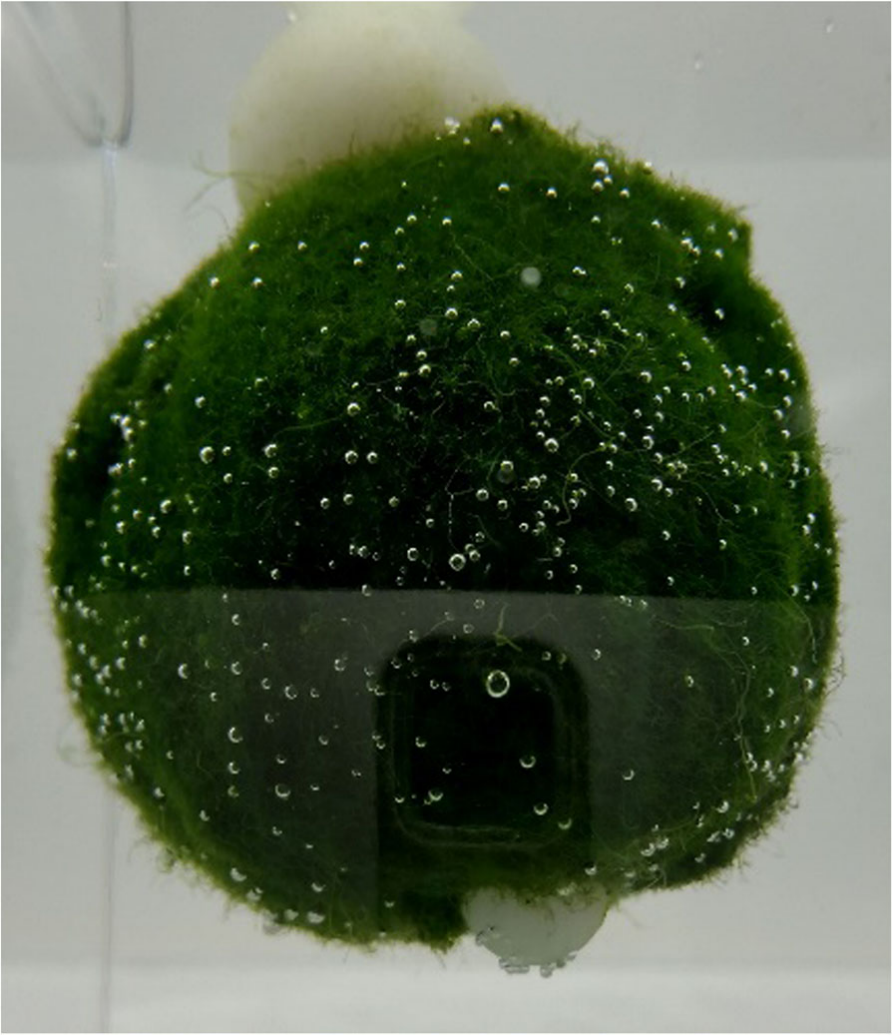
Marimo with artificially generated lower centre of mass, in order to prevent rotation. The top sphere is polypropylene, the bottom sphere is polyoxymethylene, and they are interconnected through the Marimo with a nichrome wire

The variations in movements of similar Marimo (*n*=20) were recorded (via video time lapse) under different conditions. Analysis of recorded images revealed irregular shaped Marimo floated more often than spherical shaped. It was hypothesised that their asymmetry reduces rotation allowing them to retain more bubbles. Through experimentation this characteristic was deliberately enhanced by adding positive buoyancy above the Marimo and negative buoyancy below the Marimo, to lower the centre of mass which in turn reduces rotation off the vertical axis. For best performance, the combined change in buoyancy of positive buoyancy (float), negative buoyancy (weight) and connection (link) needs to be slightly positive. The float and weight need to be free of nucleation sites to prevent additional bubble formation (which can positively bias buoyancy). This can be achieved by selecting objects with smooth surfaces, such as plastics. A Marimo modified in such a way can be seen in Fig. 4.

To measure water absorption (and quantify loss of buoyancy over time) a cohort of spheres made of different types of plastics with and without holes were weighed before and after submersion under water (see Additional file 1 for details). Testing confirmed polypropylene offers greater lift per volume than HDPE (consistency of density and water absorption were both similar). Some other types of plastics (such as polymethylpentene (TPX™) with density of 0.82 g cm ^−3^) provide greater lift per volume, but are not readily available as spheres of desired size. The float or weight can be coloured or marked to provide visual differentiation for experimentation. As the float and weight are solid (rather than hollow) they can be drilled to provided an anchor point (2 mm diameter). To secure the float and weight, whilst ensuring the Marimo is still able to float freely, a narrow (1.5 mm diameter) skewer is inserted directly from the top to bottom to ensure the float and weight are aligned with Marimo centre of mass. The available reduction in density (compared to water) of solid plastic (e.g. 0.9 g cm ^−3^ for polypropylene (PP)) is less than the available increase in density (e.g. 2.2 g cm ^−3^ for polytetrafluoroethylene (PTFE)) so the float requires a larger volume than the weight to provide an equivalent change in buoyancy. Example dimensions and material properties are shown in Table 1, however, alternative equivalent combinations are possible. An additional consideration is water absorption of the float over prolonged periods of immersion, this can be minimised through material selection (e.g. plastic made from non-polar organic species).

**Table 1:** The density and related buoyancy of various materials and Marimo

**Part**	**Material**	**Density**	**Diameter (Length)** ^∗^	**Weight**	**Buoyancy**
		**g cm** ^−3^	**mm**	**mN**	**mN**
Float (sphere)	Polypropylene (PP)	~0.9	25.4	71	7.8
Marimo (sphere)	Filamentous algae	~1.0	50 – 60	520^*†*^	-4.9
Bubbles	Gas (O_2_, CO_2_ & N_2_ mix)	0.001	0 – 5	0	0 – 20
Weight (sphere)	Polyoxymethylene (Delrin^®^)	1.42	11	9.0	-2.6
Link ^∗^	Nichrome wire	8.4	(75)	1.5	-1.3
**Combined Overall**					**−** **1** **t** **o** **+** **1** **9**

^∗^Dimensions are given as diameters for spheres and as length for the ‘link’. ^*†*^ The Marimo ball has a weight of 88mN once desiccated

### Marimo motor

When illuminated by daylight, speed of rotation is proportional to intensity. For example, approximately 0.2 rev h ^−1^ at 1 kW h m ^−2^ and approximately 0.5 rev h ^−1^ at 2.7 kW h m ^−2^. Daily illumination levels were obtained from a publicly available dataset on the photovoltaic power generation at the nearby Hamilton House, Bristol, UK. The energy of a single Marimo completing a single revolution is 0.9 mJ rev ^−1^ per Marimo, when rotating at 0.2 rev h ^−1^. This energy will increase linearly as the speed of rotation increases. The calculation can be seen in the Additional file 1.

Illumination level needed to be within a suitable range: too low and the rate of photosynthesis is too slow to generate the necessary oxygen to raise the Marimo, whereas too high level of illumination caused damage to the Marimo (as evidenced by their taking on a discoloured appearance). A PAR light sensor (SQ-120, Campbell Scientific Ltd) was located on top of the motor’s bearing support arm. The sensor’s output voltage was measured with a Fluke 8846A. For example, when illuminated by Laputa ‘60LEDS grow light’ (set to 40% of full power) the PAR sensor output was 22 mV, which corresponds to 110 *μ*mol m ^−2^ s ^−1^ (approximately 1240 lumens), which rotated the Marimo motor with an average speed of 0.25 rev h ^−1^.

### Marimo oscillator

**Fig. 5:**
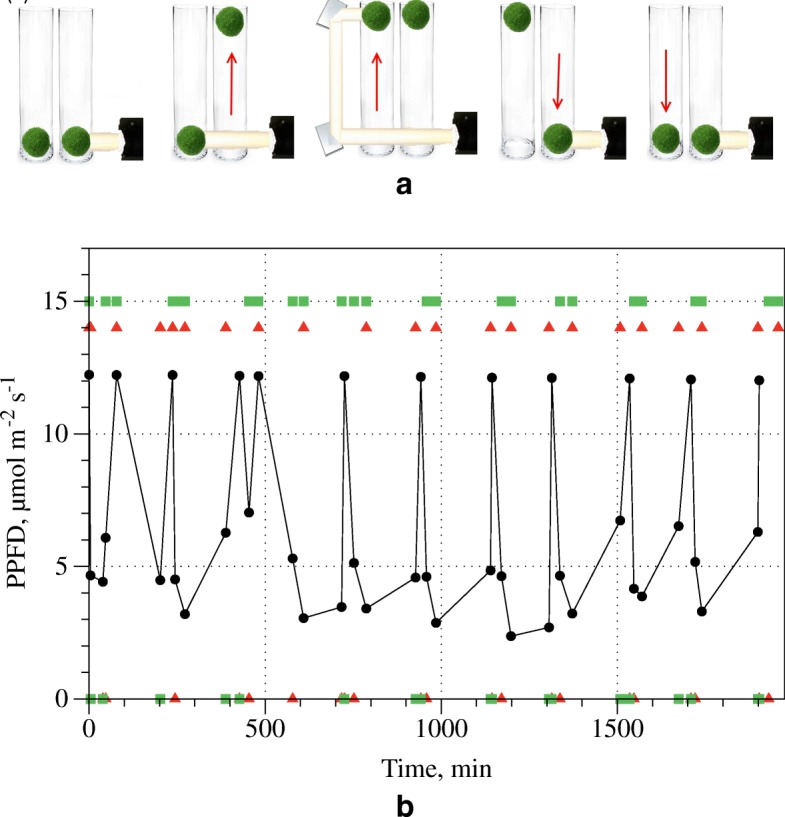
Dynamics of the Marimo oscillator. **a** Scheme of a typcial sequence of actions. **b** Level of illumination against time is shown by black circles (the connecting black line between the circles is to guide eyes). ‘Up’ and ‘bottom’ position of left (red triangle) and right (green rectangle) balls are superimposed on the plot

**Fig. 6:**
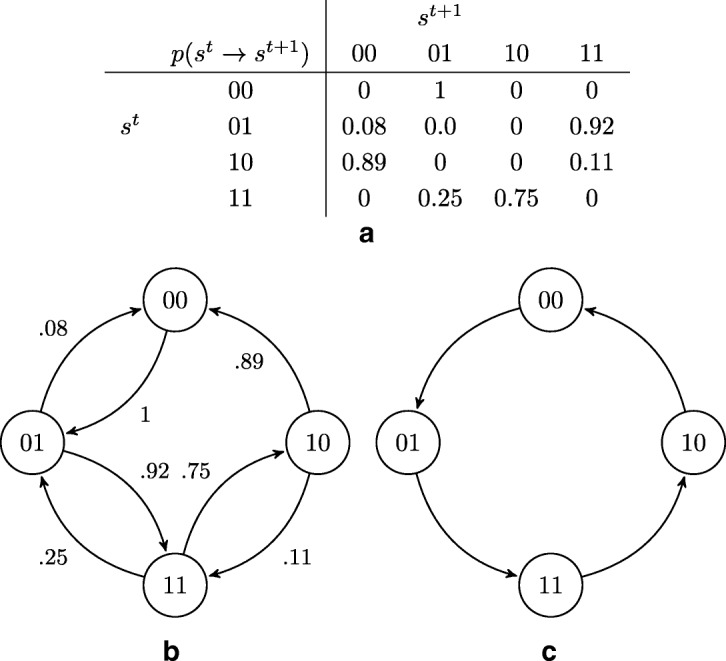
Probabilities of state transitions of a two-ball Marimo oscillator. (**a**) Experimental transition probabilities going from one state at time ‘t’ to another state at time t+1. (**b**) Probabilistic State Transition diagram, populated with values from (**a**). (**c**) Probabilistic State Transition diagram for an ideal oscillator

The Marimo balls in the experimental oscillator setup executed phases movement (Fig. 5a) detected via video recording and measurement of light passing through the water columns (Fig. 5b). Data recorded (using protocol: both Marimo low = 0 0, left low, right high = 0 1, both Marimo high = 1 1, left high, right low = 1 0) and a video of the oscillation sequence is available in the Additional files 2 and 5. Probabilities, derived from experimental recording, of transitions between states of the two-ball oscillator are given in Fig. 6a. The oscillation can be seen as a probabilistic automaton with four states (00), (01), (10), (11), corresponding to the situation of ‘left’ and ‘right’ balls being down (0) or up (1). The probabilistic state transition diagram of the automaton is shown in Fig. 6b. If only state transitions with a maximum likelihood are allowed to take place then the two-ball Marimo machine runs in the cycle shown in Fig. 6c, thus illustrating the ideal oscillator.

## Discussion

A wide range of logic gates (including: AND, NOR, OR, NAND, XOR) have been envisaged using Marimo with light as ‘input signal’, ‘output signal’ and ‘actuation power’. In other words, depending on the desired functionality, light is interchangeable between all three functions [input signal (data), output signal (data), actuation power (energy)]. This provides potentially zero electric grid consumption when operating on sunlight. Further, processing based on variable density units can be both modular and scalable. A key benefit to the devices presented is, therefore, the efficiency inherent in utilising photosynthetic (i.e. carbon- capturing), self-growing, solar-powered devices whose running costs are essentially null, regardless of whether their application lies in creating unconventional computing devices (logic gates, oscillators) or engineering (motor, e.g. applied to electricity generation).

**Fig. 7:**
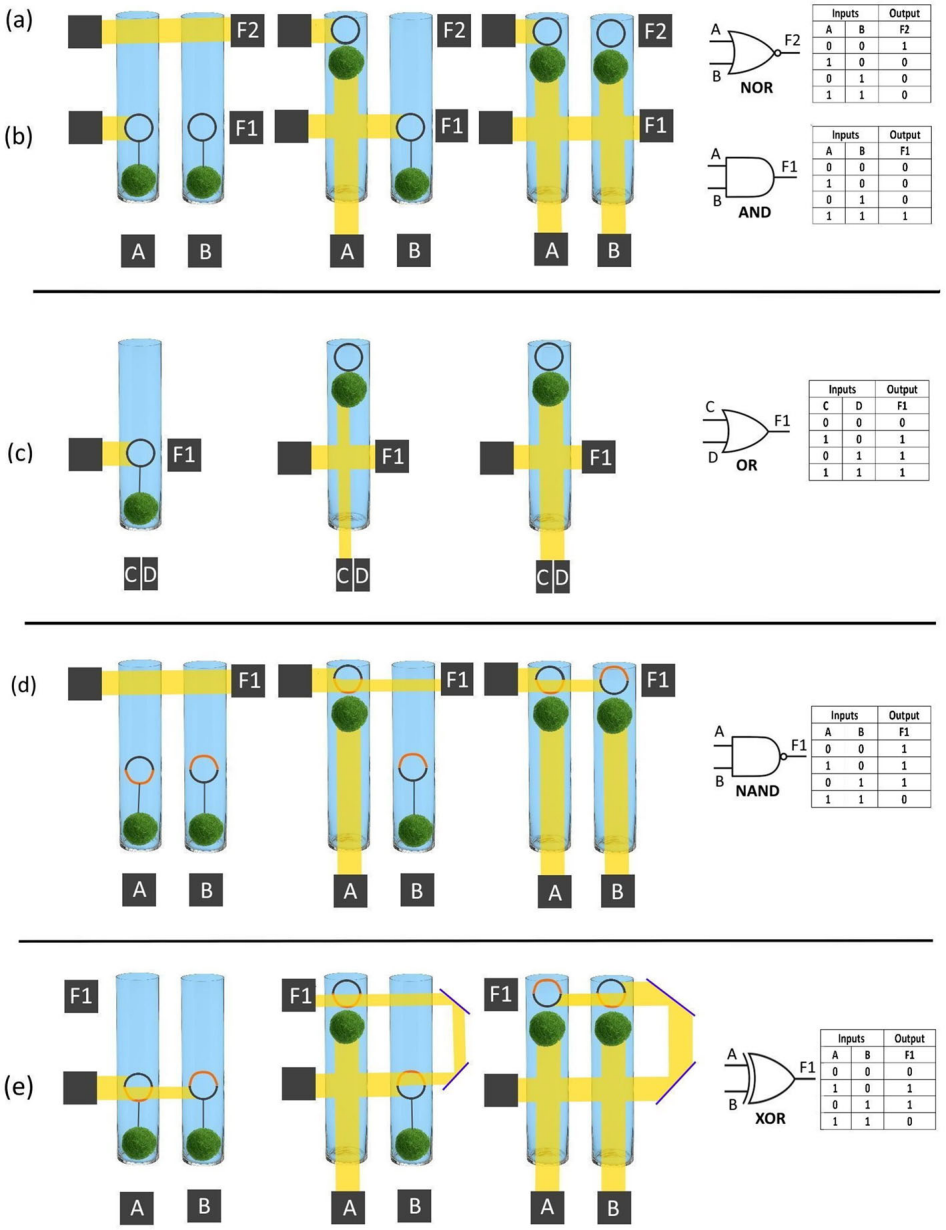
Logic gates based on Marimo: (**a**) NOR (**b**) AND (**c**) OR (**d**) NAND (**e**) XOR. The Marimo are represented with green balls. The floats are represented by orange and/or black circles. The black section of a float is opaque, while the orange section is transparent. The input signals are shown as A, B, C, or D. The output signal is observed by sensor F1 or F2. An ‘always-on’ light sources is portrayed by a black box

In order to extend the systems’ capabilities into logic gates, a refined set up has been designed. The layouts for these gates can be seen in Fig. 7. The main alteration is that the float has transparent and opaque sections, as required. Inputs are represented by the presence or absence of a light beam entering the system, and outputs by a light beam exiting the system. Such a system could also be implemented using other vertically-controlled units [17–19].

During the development of the Marimo logic gates it was observed that Marimo can hold a stable position between the ‘top’ and ‘bottom’ of the containment vessel for prolonged periods (upwards of 30 min, video available in the Additional file 4), with a suitable lighting configuration. This enables the potential advancement of logic gates to include multi-state operations. Further, by graduating the light level, an analogue system can be conceived.

It was observed that Marimo often take a period (several hours) before forming bubbles and movement in a new/refreshed environment. This induction period may be a combination of several factors including: rising concentration of dissolved gases in local water before bubble formation, re-acclimatisation of the organisms to the rapid change in environment, and the state of the Marimos’ photosynthetic systems prior to beginning the experiment [21].

The 1D rotational movement (of Marimo motor) can be extended to 2D movement. One can envisage such a system whereby multiple Marimo are located inside individual transparent chambers (spheres), which are themselves located near the inside surface of a larger partly transparent sphere. Marimo exposed to illumination will generate gas (via photosynthesis) creating positive buoyancy. The net change in buoyancy will rotate the larger sphere (rover) away from the light source. Each of the smaller spheres would contain a gas vent (located at the largest distance from centre of larger sphere) such that the gas can escape when the smaller sphere moves to the ‘top’ of the larger sphere. It is worth noting that because the surface area of a sphere increases to the square of its radius, the potential power of a Marimo rover would increase quadratically with its size. Potentially, such a light-powered Marimo rover could autonomously travel on land or in water (if outer sphere is suitably configured), and navigate topology. The topology of the land could be inferred by remotely tracking the movement of the rover (e.g. add RF reflector and track via satellite). A prototype rover is under construction and will be reported on in the future.

Potential applications of actuators and individual logical gates based on Marimo can be found in situations where speed of operation is not essential, but device longevity is. For example, a logical device such as the one presented here could be used as a light-sensitive controller for a microbial fuel cell system, such as the one presented in Ref. [22]. The rationale behind this approach is that such a controller — which could be effective for the decades-long lifespan of the Marimo used and would not require an electrical power source — would switch between power usage or storage based on the user’s diurnal energy demands. Furthermore, any low speed of operation could be mitigated in the various applications of Marimo motors, such as the aforementioned electricity generation. If, for example, Marimo motors were used to generate electricity via induction from only sunlight input, device efficiency could be improved through scaling and design optimisation. Whilst electricity generation could not be expected to be enormous through these means, we consider the concept worthy of further investigation as a current opinion on alternative fuel sources is that a variety of ‘green’ methods should be utilised in combination to achieve better climate outcomes. As such, certain perceived ‘failings’ of Marimo-based devices may be mitigated by factors such as their ability to capture carbon and not requiring heavily-refined or enriched materials in their production, as is the case for solar panels.

The potential speed of operation is an important consideration in biomimetic designs, as it is comparable to data processing and actuation time. While most plants move relatively slowly (compared to animals), some have evolved fast responses. For example, the Venus flytrap (*Dionaea muscipula*) can capture a fly by closing its trap (a modified leaf) in less than one tenth of a second when triggered (physical contact with plant’s hairs) [23]. Therefore, the speed of operation cannot be considered to be limited solely due to its biological nature. Whilst the current investigation did not examine variation in Marimo diameter, this would be an important aspect to focus on in future studies as there could be a relationship between the size of a Marimo and its movement.

Although the applications outlined above are proposed specifically in applications where slow speed of operation is not an impediment, the principles of operation outlined in this paper can be applied to a smaller scale which could be used, in turn, to enhance the operation speed. As photosynthesis is a characteristic of multiple species of plants, algae, bacteria and protozoa, the limitation on the size of a living oxygen-generating buoyancy system is therefore bounded by the physical dimensions of a single photosynthetic cell plus a surrounding medium for capturing any oxygen generated, although smaller systems may be considered as viable through the use of decellularised chloroplasts. We propose that, if immobilised in a suitable porous, water-stable, light-permeable substance such as sodium alginate, small colonies of microalgae (e.g. *Micromonas pusilla*, approximately 3 *μ*m in length [24]) or cyanobacteria (approximately 1 *μ*m in length) could be utilised in much the same manner as the previously described Marimo devices. The use of microfluidic technology has been demonstrated as a reliable method for generating alginate beads with embedded substances in the range of 10 *μ*m [25], hence we propose this to be a realistic estimate of the size of a single photosynthetic, variable-density buoyancy system, for use in miniaturised versions of the Marimo devices previously described, which could be engineered to have much faster operation times through exploiting lower-mass components and shorter travelling distances for the photosynthetic elements.

## Conclusion

We have demonstrated that a diverse range of devices can be built using Marimo. Our systems act as (1) biosensors, in that their output varies with external environmental conditions; (2) actuators, in that we have constructed a Marimo-powered rotational motor; (3) and logic gates, using the natural vertical motions of Marimo we have established the first Marimo oscillator and proposed schematics for a wide range of logic gates based on the time-varied density of Marimo. The performance of these example systems has been enhanced by optimisation of the centre of mass and overall buoyancy of individual Marimo. The principle of using photosynthesis to control operations has been demonstrated. The potential to extend functionality to a autonomous, light-powered rover has been proposed. Transferring the principles of operation to the cellular scale would enable greater processing density. Opportunities for further enhancements to functionality have been identified and are under development.

## Additional files

Additional file 1 Additional supplementary information is available in an online file. This includes construction information on the motor and balancing rigs, details on the floats used, and details on the light sources. (PDF 1806 kb)

Additional file 2 Video of the Marimo Oscillator in action. (MP4 13,077 kb)

Additional file 3 Video of the Marimo Motor in action. (MP4 12,292 kb)

Additional file 4 Video of the Marimo whilst vertically stable. (MP4 12,592 kb)

Additional file 5 Processed data of the Marimo oscillator video. (XLSX 12 kb)

## Data Availability

The datasets made and analysed during the current study are available from the corresponding author on reasonable request.
