# Supplementary material for: Marimo machines: oscillators, biosensors and actuators
Source: J Biol Eng. 2019 Sep 3;13:72. doi: 10.1186/s13036-019-0200-5 (PMC6724321; doi:10.1186/s13036-019-0200-5)
Supplement: Supplementary file 1 — Additional supplementary information is available in an online file. This includes construction information on the motor and balancing rigs, details on the floats used, and details on the light sources. (PDF 1806 kb) [file 13036_2019_200_MOESM1_ESM.pdf]

Electronic Supplementary Information

Marimo Machines:
Oscillators, Biosensors and Actuators

Neil Phillips[1,*], Thomas C. Draper[1], Richard Marne[1,2], and Andrew Adamatzky[1]

[1]Unconventional Computing Laboratory, University of the West of England, Bristol, BS16 1QY, UK
[2]Department of Applied Sciences, University of the West of England, Bristol, BS16 1QY, UK
[*]Corresponding author: Neil.Phillips@uwe.ac.uk

S1 Motor frame

The rotor of the marimo motor was (magnetically) suspended on bespoke, compact frame to fit inside water tank (aquarium). The dimensions of the frame were: base 240 mm wide x 290 mm length and support arms 220 mm tall x 240 mm wide. The frame was constructed from anodised aluminium section (30 mm × 30 mm) with integrated 'T' slots. The sections were bolted together. Rubber feet were added to four corners. The neodymium magnets were grade N35 (30mm diameter and 10 mm thickness). One magnet was mounted on a screw thread to allow precise adjustment of the gap between the magnets, see Fig. S1. The shaft was mild steel, 8 mm diameter and 100 mm length (tip of cone to flat end).

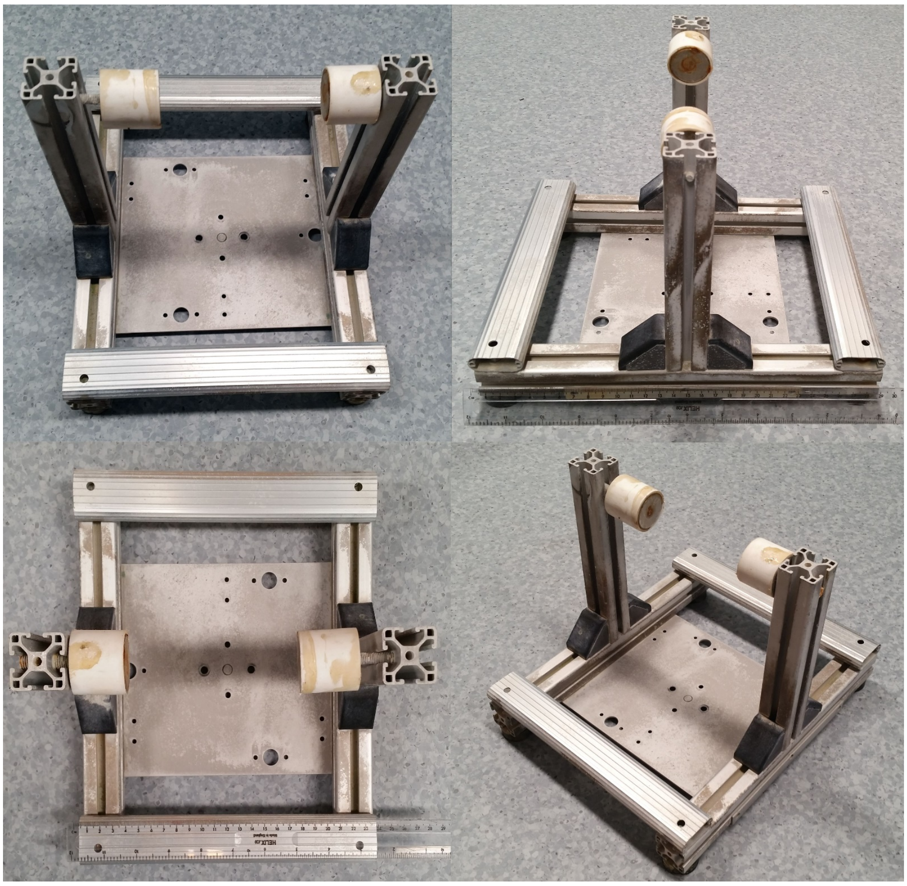

Figure S1: Motor frame used inside water tank

S2 Rotor balance rig

A bespoke rig was built to balance the rotor of Marimo motor. The dimensions of the (collapsible) frame were: base 250 mm wide x 750 mm length and support arms 400 mm tall with adjustable width 0 to 500 mm. The frame was constructed from anodised aluminium section (40 mm × 40 mm) with integrated 'T' slots. The sections were bolted together. Rubber feet were added to four (folding) legs. Four neodymium magnets (grade N35) were stacked on both sides forming magnets of 30 mm diameter and 40 mm thickness. One magnet was mounted on a screw thread to allow precise adjustment of the gap between the magnets, see Fig. S2.

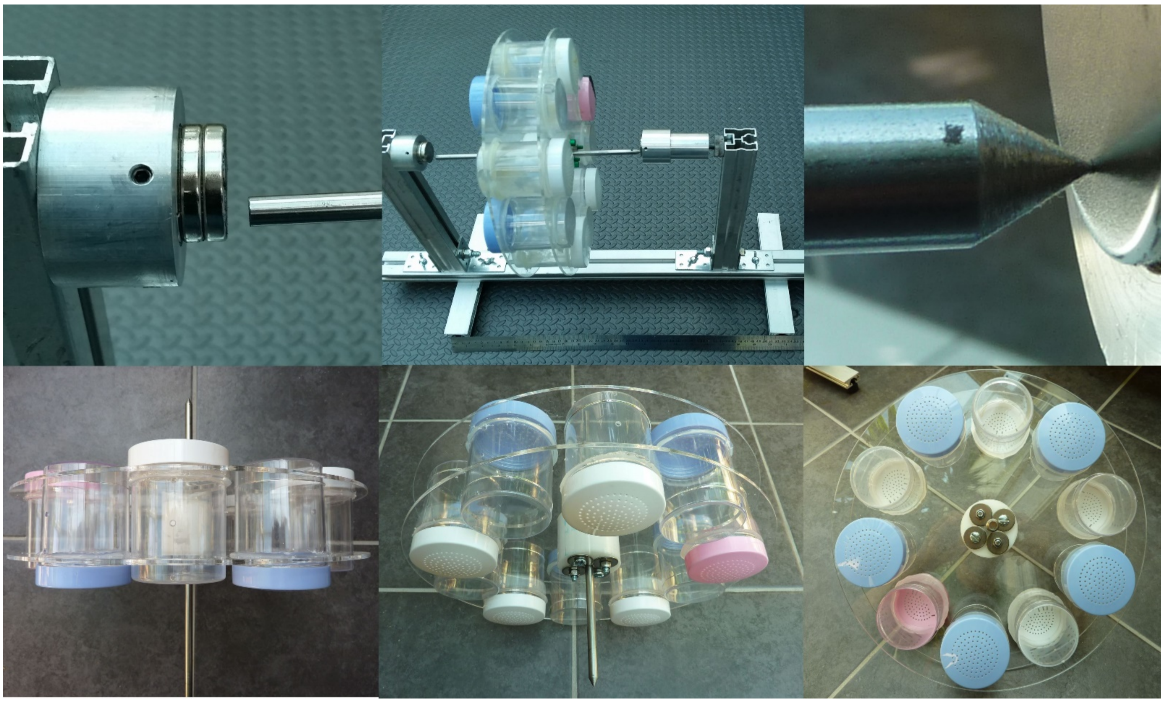

Figure S2: Rotor balance rig

The motor frame could not support the rotor out of the water and balancing in the water was impractical. Consequently, a balancing and operating rig were required. The balance rig required stronger magnets than the motor frame as rotor weight was not reduced by positive buoyance (in water). The rotor was balanced by manually adding weight to the acrylic disc until the out-of-balance was less than 50 g mm.

S3 Marimo Motor power output calculations

When illuminated, the Marimo photosynthesise and produce oxygen bubbles. These bubbles, due to their density being lower than that of water's, rise and become trapped against the inside surface of the individual cylinder containing the Marimo. When the cylinder reaches to the top of its rotation the bubbles are able to escape, permitting rotation of the motor to continue. During rotation, the bubbles in each cylinder rise by 250 mm.

The average volume of gas generated by a Marimo is approximately 3.5 cm^3 d^{-1}. With a rotational speed of 0.2 rev h^{-1}, half a rotation takes 2.5 h and 0.36 cm^3 of gas is generated. To calculate the lift, the mass of the (rising) gas bubbles is subtracted from the mass of the water 'sinking' (becoming displaced):

$$(1.00\,\mathrm{g\,cm^{-3}} \times 0.36\,\mathrm{cm^3}) - (0.001\,\mathrm{g\,cm^{-3}} \times 0.36\,\mathrm{cm^3}) = 0.36\,\mathrm{g}. \quad (1)$$

Potential energy (PE) can be expressed as PE $= mgh$, where m is mass, g is acceleration due to gravity, and h is the distance displaced (or height). Therefore,

$$\mathrm{PE} = mgh = 3.6 \times 10^{-4}\,\mathrm{kg} \times 9.81\,\mathrm{m\,s^{-2}} \times 0.25\,\mathrm{m} = 0.9\,\mathrm{mJ}. \quad (2)$$

The resulting value is based on a single Marimo completing one revolution, therefore the energy can be more properly displayed as 0.9 mJ Marimo^{-1} rev^{-1}, when rotating at 0.2 rev h^{-1}.

S4 Comparison and construction of floats

Table S1: The weights of floats tested for use with Marimo, both before and after drilling and before and after submersion in water for 72 h. The diameter of all spheres was 25 mm. High density polyethylene (HDPE) and polypropylene (PP) were tested. All measurements were taken on an analytical balance.

	**Dry (no hole)**	**72 h Submersion (no hole)**	**Dry (2 mm hole)**	**72 h submersion (2 mm hole)**
	**g**	**g**	**g**	**g**
**HDPE**				
**I**	7.6928	7.6938	7.6407	7.6472
**II**	7.7024	7.7042	7.6544	7.6583
**III**	7.7091	7.7104	7.6735	7.6777
**IV**	7.7224	7.7230	7.6931	7.7037
**V**	7.7300	7.7310	7.6877	7.6937
**VI**	7.7600	7.7605	7.7197	7.7275
**Average Weight**	7.7195	7.7205	7.6782	7.6847
**Average Absorption**		0.0010		0.0065
**PP**				
**A**	7.2201	7.2202	7.1874	7.2034
**B**	7.2159	7.2166	7.1463	7.1500
**C**	7.2461	7.2472	7.1928	7.2011
**D**	7.2575	7.2591	7.2227	7.2274
**E**	7.1793	7.1795	7.1320	7.1363
**F**	7.2356	7.2358	7.2080	7.2149
**Average Weight**	7.2258	7.2264	7.1815	7.1889
**Average Absorption**		0.0007		0.0073

S5 Bubble volume by light level

Table S2: The amount of gas produced by individual Marimo balls exposed to natural light. Daily variance in lighting was measured via the output of a local PV system.

**Date**	**Ambient light level**	**Daily volume of bubbles released / ml**					
	**Local PV output / W h m^{-2}**	**Control**	**Marimo A**	**Marimo B**	**Marimo C**	**Marimo D**	**Average**
18/11/2017	14	0	1.5	1.5	0	0.5	0.875
19/11/2017	62.9	0					
20/11/2017	17.6	0	1	1	0	0	0.5
21/11/2017	33.7	0					
22/11/2017	27.2	0	1	2.5	2	2	1.875
23/11/2017	58.5	0					
24/11/2017	52.8	0	2	2	1	0.5	1.375
25/11/2017	70.5	0					
26/11/2017	49.2	0					
27/11/2017	45.8	0	6	6	2.5	2.5	4.25
28/11/2017	54.5	0	4	5	2	1	3
29/11/2017	39.5	0	3	5	2	1	2.75
30/11/2017	40	0	3	3	1	1	2
01/12/2017	48.9	0	4.5	3.5	1.5	1	2.625

S6 Illumination levels

A range of lamps were used during experimentation. Illumination against distance for each type of lamp is shown in table S3. Photosynthetically Active Radiation (PAR) light sensor (Campbell Scientific Ltd, model SQ-120, measurement repeatability: < 1 %, linear response: 5 μmol m^{-2} s^{-1} mV^{-1}) was used to measure illumination levels. The output voltage of the sensor was measured with a Fluke 8846A precision multimeter.

Photosynthetic Photon Flux Density (PPFD) rather than lux was recorded, as the PAR sensor measures how many photons (within the portion of the spectrum useful for photosynthesis) are striking the surface. Every photon is counted the same. By contrast, a lux sensor measures light in the visible spectrum and weights the light best seen by human eyes. Lux sensors tend to measure lower than PAR/PPFD sensors. Conversion of PPFD (μmol m^{-2} s^{-1}) to lux is achieved using the manufacturers instructions.

The Marimo oscillator was tested using a Martin Rush PAR 1 70W RGBW Cree LED Lamp. Beam angle 20 degrees. Independent & variable power on primary colours (red, blue & green): 0 to 255 (0 to 100 %). The logic testing used a bespoke lamp. 10 W LED array (660 nm) was mounted on fan cooled heatsink, see Fig. S3(a). The optics were adapted from a LEDSPOT-3WW spot lamp (Pulse Audio Ltd) and configured to work with LED array and heatsink. A beam focus of 4 degrees was achieved, see Fig. S3(b). Motor testing used variable power, 300W, full spectrum, plant growth lamp, brand name LAPUTA, model number '60LEDS grow light'. The power output of all lamps can be seen in table S3.

Table S3: Illumination levels of lamps in air. *Red & blue at 100 %, green at 0 %. †Red & blue at 18 %, green at 0 %. ‡Our bespoke lamp operated solely at 660 nm. $^{\alpha}$ location first Marimo in oscillator. $^{\beta}$ location second Marimo in oscillator. $^{\gamma}$ average illuminated Marimo in motor. Illumination levels attenuated by water.

**Distance (mm)**	**Martin Rush PAR 1* 70W**	**Martin Rush PAR 1† 70W**	**Bespoke‡ 10W**	**LAPUTA 60LEDS 300W**
0	650	56	158	$\approx$ 1000
100	510	50	124	$\approx$ 455
200	429	40	80	$\approx$ 364
300	382	37	68	$\approx 300^{\gamma}$
400	345	33^{α}	39	242
500	314	27^{β}	23	186
600	248	19	16	160
700	207	14	12	131
800	156	11	9	110
900	122	8	7	91
1000	72	7	6	79

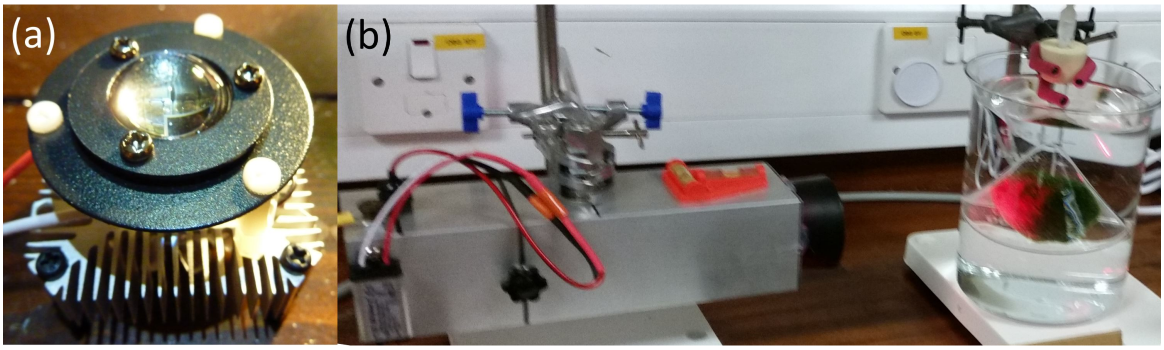

Figure S3: Bespoke lamp (a) LED array and lens on heatsink (b) narrow (4 degree) light beam

S7 Other Supporting Files

Included in the electronic supplementary information (ESI) are videos portraying the Marimo in action. A description of these videos can in found in table S4.

Table S4: Descriptions of the videos included in the ESI.

File Name	Description
Oscillator.mp4	A time-lapse video of the Marimo oscillator
Motor.mp4	A time-lapse video of the Marimo-powered motor
Hover.mp4	A time-lapse video of a vertically stationary Marimo
Oscillator_data.xlsx	Processed data from the Oscillator video
